# Abatacept for steroid-refractory fulminant immune checkpoint inhibitor myocarditis with triple M overlap syndrome: a case report with serial endomyocardial biopsy assessment

**DOI:** 10.1093/ehjcr/ytag620

**Published:** 2026-08-30

**Authors:** Ruriko Matsui, Kimi Sato, Kazuko Tajiri, Tomoko Ishizu

**Affiliations:** Department of Cardiology, Institute of Medicine, University of Tsukuba, Tennodai, Tsukuba, 3058575, Japan; Department of Cardiology, Institute of Medicine, University of Tsukuba, Tennodai, Tsukuba, 3058575, Japan; Department of Cardiology, National Cancer Center Hospital East, Kashiwanoha, Kashiwa, 2778577, Japan; Department of Cardiology, Institute of Medicine, University of Tsukuba, Tennodai, Tsukuba, 3058575, Japan

**Keywords:** Immune-related adverse event, Triple M overlap syndrome, Immune checkpoint inhibitor (ICI)-related myocarditis, Endomyocardial biopsy, Abatacept, Case report

## Abstract

**Background:**

Triple M overlap syndrome is a rare, but often fatal, immune-related adverse event. Evidence on the effectiveness of immunosuppressive treatments, including abatacept, and disease monitoring in complex cases remains limited.

**Case Summary:**

A man in his 60s developed fulminant immune checkpoint inhibitor (ICI)-related myocarditis after nivolumab plus ipilimumab for recurrent lung adenocarcinoma, complicated by triple M overlap syndrome (myocarditis, myositis, and myasthenia gravis). Despite high-dose corticosteroids and intravenous immunoglobulin, cardiac biomarkers worsened, with progressive conduction abnormalities and a rapid decline in the left ventricular ejection fraction to 23%. Upon transfer to our institution, he developed sustained ventricular tachycardia, requiring direct current cardioversion and intra-aortic balloon pump support. Endomyocardial biopsy (EMB) at admission showed CD8^+^ T-cell-predominant myocarditis, consistent with a fulminant, steroid-refractory disease course. Abatacept was initiated after a multidisciplinary discussion, resulting in haemodynamic and arrhythmic stabilization. Guided by serial EMB findings of residual myocardial inflammation, abatacept was administered four times by hospital Day 30, along with intravenous methylprednisolone pulse therapy, plasma exchange, intravenous immunoglobulin, and tacrolimus. Left ventricular ejection fraction gradually improved to 56%, and a follow-up EMB on Day 88 demonstrated marked histopathological recovery.

**Discussion:**

This case illustrates that abatacept-based multidisciplinary therapy may help stabilize fulminant steroid-refractory ICI-related myocarditis with triple M overlap syndrome. Serial EMB provided a histopathological assessment of residual myocardial inflammation and guided immunosuppressive therapy, suggesting a potential role for serial biopsy in complex cases.

Learning points

- Abatacept-based multidisciplinary therapy may help stabilize fulminant steroid-refractory ICI-related myocarditis with triple M overlap syndrome.
- Serial EMB provided a histopathological assessment of residual myocardial inflammation and guided immunosuppressive therapy, suggesting a potential role for serial biopsy in complex cases.

## Introduction

Increased use of immune checkpoint inhibitors (ICIs) has been accompanied by a growing recognition of immune-related adverse events (irAEs); ICI-associated myocarditis is an uncommon but highly lethal complication. Neuromuscular toxicities associated with ICIs have emerged as important causes of morbidity and mortality. The simultaneous occurrence of myocarditis, myositis, and myasthenia gravis (MG), known as triple M overlap syndrome, represents a severe irAE phenotype.^1–3^ This syndrome is characterized by rapid clinical deterioration, respiratory failure, malignant arrhythmias, cardiogenic shock, and a high in-hospital mortality rate of 40%.^3^ Although current recommendations advocate the prompt initiation of high-dose corticosteroids for ICI myocarditis,^4^ evidence-based treatment strategies for steroid-refractory cases remain limited. Abatacept, a cytotoxic T-lymphocyte antigen 4 (CTLA-4) agonist, has emerged as a therapeutic option for severe ICI myocarditis. Nevertheless, the optimal dose, timing, duration, and monitoring strategy for abatacept therapy have yet to be established, particularly in patients with concomitant triple M overlap syndrome.

Here, we report a case of steroid-refractory fulminant ICI myocarditis complicated by triple M overlap syndrome following nivolumab and ipilimumab therapy. Repeated administration of abatacept as part of multimodal immunosuppressive therapy was associated with substantial clinical recovery. Importantly, serial endomyocardial biopsy (EMB) findings provided histopathological evidence of persistent and subsequent resolution of myocardial inflammation, thereby supporting therapeutic decisions throughout the patient’s clinical course.

## Summary figure

**Figure:**
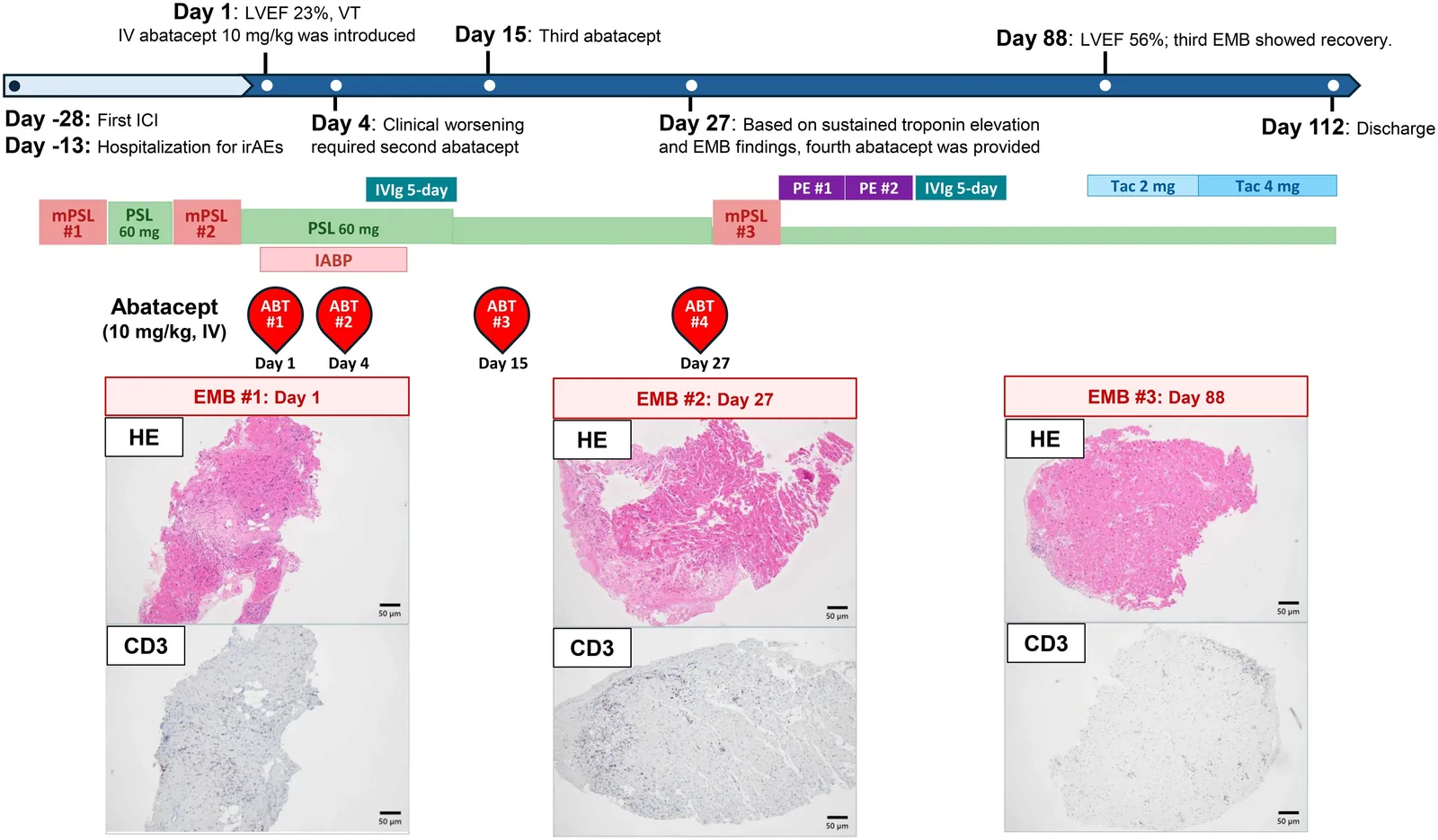


## Case presentation

**Figure 1:**
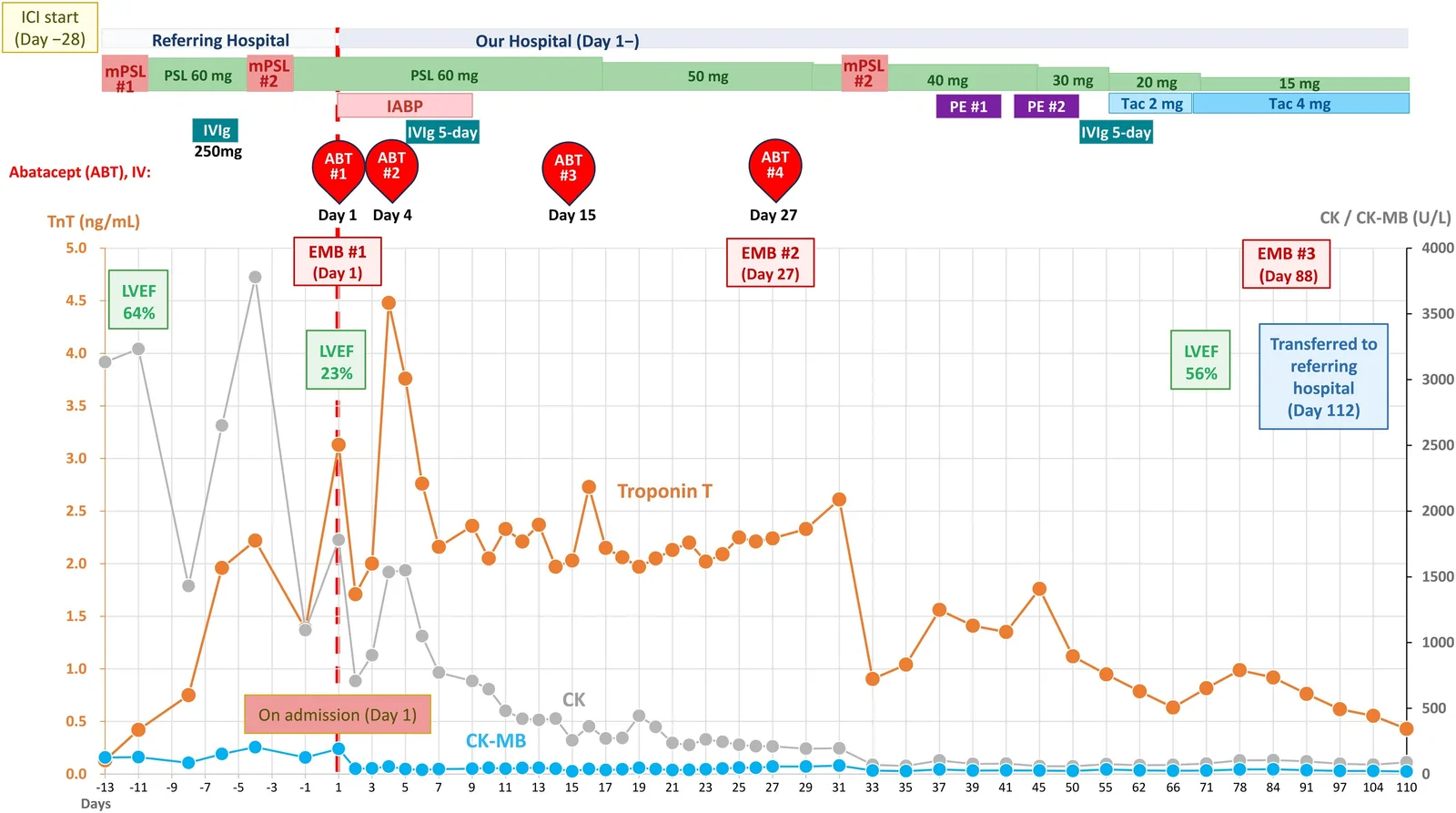
Clinical course. Abbreviations: PSL, prednisolone; TAC, tacrolimus; TnT, troponin T; ICI, immune checkpoint inhibitor; mPSL, methylprednisolone pulse; IVIg, intravenous immunoglobulin; ABT, abatacept; EMB, endomyocardial biopsy; IABP, intra-aortic balloon pump; PE, plasma exchange; LVEF, left ventricular ejection fraction.

A man in his 60s was transferred to our hospital with worsening ICI-related myocarditis and MG. The patient had a cardiovascular history of diabetes mellitus and a myocardial infarction 17 years prior, which was treated with percutaneous coronary intervention to the left anterior descending and right coronary arteries. Two years prior to admission, he was diagnosed with lung adenocarcinoma in the right lower lobe and underwent lobectomy followed by adjuvant chemotherapy with vinorelbine plus cisplatin. After completing adjuvant chemotherapy, a distant recurrence in the left lung was treated with left upper segmentectomy. He remained free of recurrence until a new lesion in the right lung was detected 2 months prior to transfer. Combination ICI therapy with nivolumab and ipilimumab was subsequently initiated, with the first infusion administered 28 days before transfer. Twelve days after ICI initiation (Day −16), he developed progressive limb weakness, diplopia, and dysphagia, leading to admission at the referring hospital on Day −13 for suspected irAEs. While he had no cardiovascular symptoms and echocardiography showed preserved left ventricular ejection fraction (LVEF) of 64%, his troponin T (TnT) level was elevated to 0.13 ng/ml, creatine kinase (CK) was markedly elevated at 3133 U/L, and electrocardiography (ECG) showed a newly developed right bundle branch block (*Figure 1*). According to the 2022 ESC Cardio-Oncology diagnostic criteria for ICI-associated myocarditis,^4^ elevated TnT together with two minor criteria—the clinical syndrome (including muscle weakness and diplopia) and new conduction system disease (new-onset right bundle branch block)—met the clinical diagnostic criteria for ICI-associated myocarditis. The marked CK elevation, along with muscle weakness, supported concomitant ICI-associated myositis, and MG was also clinically diagnosed at the referring hospital. Taken together, these findings confirmed concurrent myocarditis, myositis, and MG, establishing the diagnosis of triple M overlap syndrome. He received two courses of high-dose methylprednisolone pulse therapy (1000 mg/day for 3 days) and one cycle of intravenous immunoglobulin (IVIg; 250 mg for 1 day); however, cardiac biomarkers continuously elevated alongside evolving ECG abnormalities. The patient was transferred to our hospital for further management.

**Figure 2:**
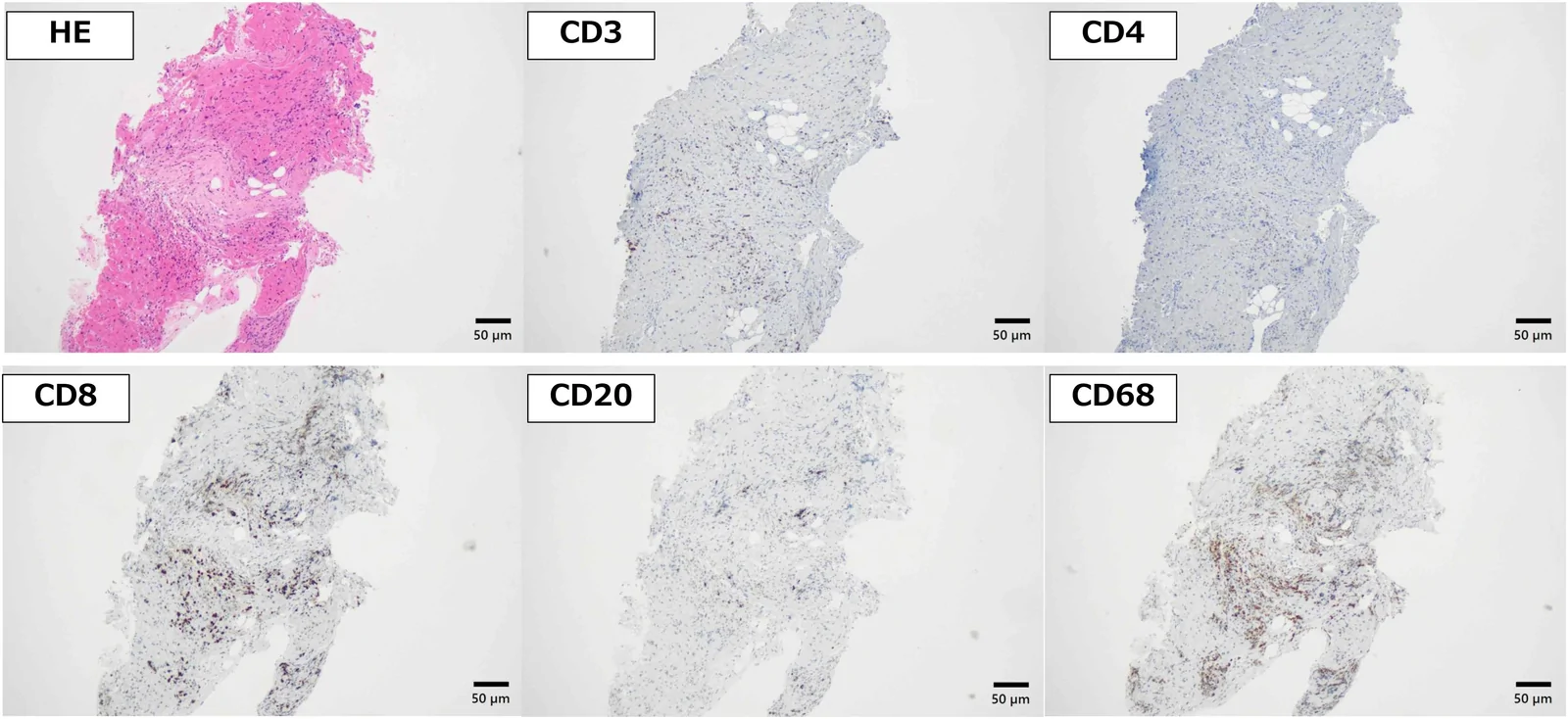
Histopathological findings on admission. Endomyocardial biopsy conducted upon admission revealed dense inflammatory cell infiltration with myocyte necrosis. CD8^+^ cytotoxic T cells are predominant among CD4^+^ helper T cells. A marked increase is noted in the number of CD68^+^ macrophages, and a mild increase is observed in the number of CD20^+^ B cells.

On admission (Day 1), the patient presented with progressive dyspnoea and cardiogenic shock; TnT was markedly elevated at 3.13 ng/ml (institutional reference range, <0.10 ng/ml), with CK 1781 U/l (reference range, 63–257 U/l) and CK-MB 192 U/l (reference range, <10 U/l). ECG showed an alternating bundle branch block with a Sokolow-Lyon QRS voltage of 0.49 mV. Echocardiography showed marked deterioration in LV function, with an LVEF of 23%, representing a rapid progression of myocarditis. Applying the novel risk score proposed by Power *et al*.^5^ yielded a score of 4 (presence of cardiomuscular symptoms, Sokolow-Lyon voltage ≤0.5 mV, LVEF <50%, and TnT >20-fold upper limit of normal). Owing to haemodynamic instability, intra-aortic balloon pump (IABP) was introduced, and urgent cardiac catheterization was performed. Coronary angiography showed multivessel stenosis, consistent with known coronary artery disease; however, these findings did not fully explain the severity and rapid progression of myocardial dysfunction. During angiography, an alternating bundle branch block degenerated into a sustained ventricular tachycardia (VT), requiring direct-current cardioversion and intravenous amiodarone and lidocaine. Subsequently performed EMB demonstrated diffuse CD3^+^ T-lymphocyte infiltration, predominantly CD8^+^, along with CD68^+^ macrophage infiltration and myocyte injury, consistent with fulminant ICI myocarditis (*Figure 2*).

**Figure 3:**
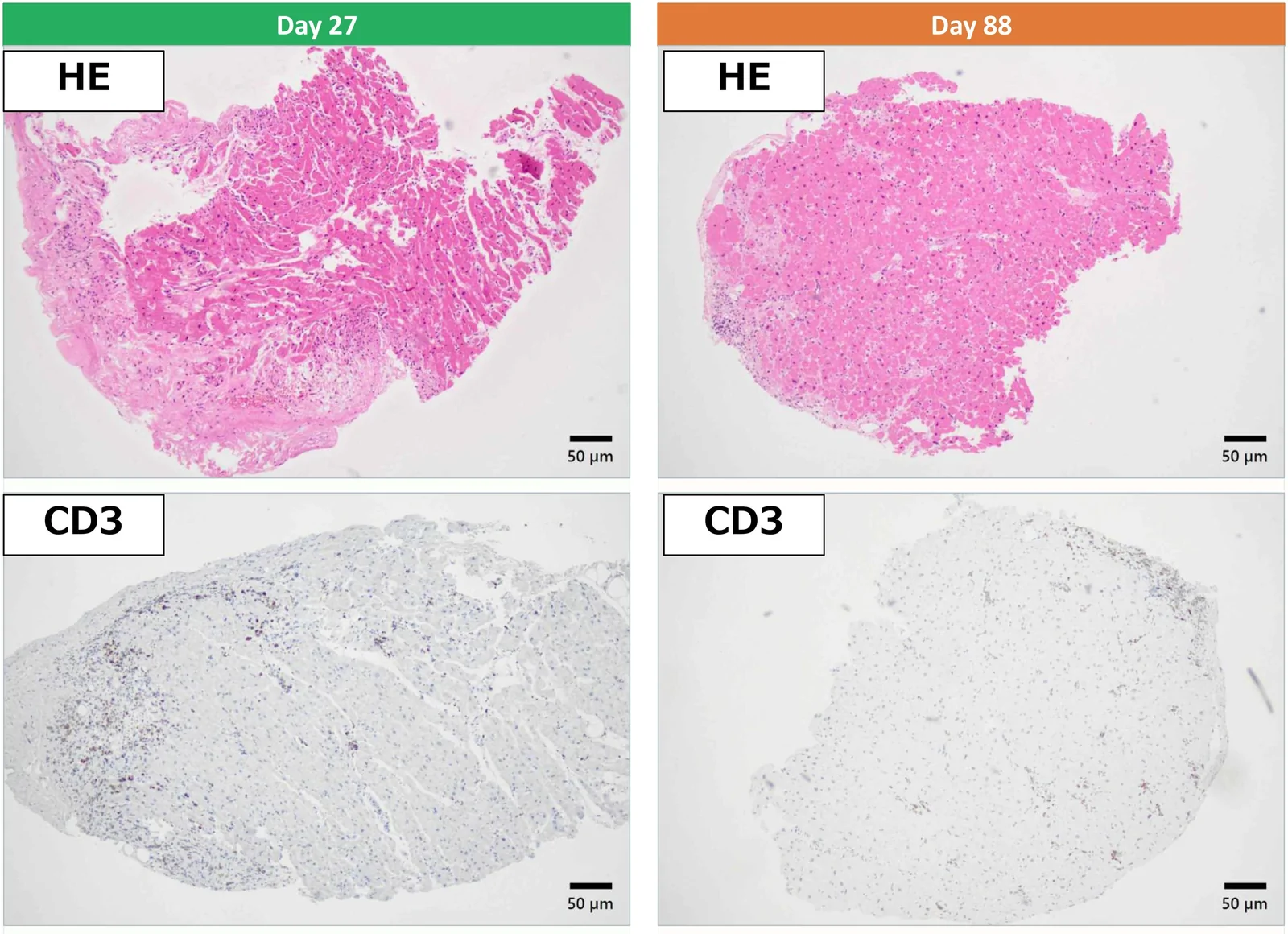
Histopathological findings during follow-up. Subsequent endomyocardial biopsy on Day 27 revealed reduced inflammation with residual lymphocytic infiltrates. On Day 88, mild inflammation persisted despite clinical improvement.

High-dose intravenous methylprednisolone pulse therapy, oral corticosteroids, and intravenous immunoglobulin failed to control the disease activity. A multidisciplinary discussion involving cardiology, oncology, neurology, and rheumatology led to the decision to start intravenous abatacept 750 mg (10 mg/kg). Following the first dose on Day 1, the VT and conduction abnormalities resolved immediately, and the TnT decreased to 1.71 ng/ml. However, recurrence of conduction abnormalities on ECG, increased premature ventricular contractions, and a re-elevation of TnT to 4.4 ng/ml on Day 4 indicated ongoing myocarditis activity, prompting a second dose of abatacept. After the second abatacept dose on Day 4 and subsequent IVIg (25 g/day for 5 days), the TnT decreased to ∼2.5 ng/ml, and the IABP was successfully removed on Day 9. However, TnT remained persistently elevated at ∼2.0–2.5 ng/ml without further decline, prompting a third dose of abatacept on Day 15, considering its half-life of approximately 13 days. Repeat EMB on Day 27 showed CD8^+^ T cell-predominant infiltration with CD68^+^ macrophages, albeit with partial attenuation (*Figure 3*, see Supplementary material online, *Figure S1*). Together with the cardiac biomarker trend, residual myocardial inflammation on EMB prompted a fourth dose of abatacept on Day 27, followed by a third course of methylprednisolone pulse therapy (1000 mg/day for 3 days), plasma exchange, and IVIg (25 g/day for 5 days). Tacrolimus was administered during corticosteroid tapering.

EMB on Day 88 showed a marked reduction in inflammatory cell infiltration compared to baseline, supporting the histological evidence of disease stabilization. Cardiac biomarkers gradually returned to near-normal levels, and no further fatal arrhythmias were observed. The LVEF recovered to 56% on follow-up echocardiography. Corticosteroid doses were gradually tapered with concomitant tacrolimus administration without myocarditis recurrence. Throughout the clinical course, no significant treatment-related adverse events were observed, including bleeding, infection, or procedure-related complications associated with serial EMB. The patient was subsequently transferred to a referral hospital on Day 112 for cardiac rehabilitation and management of MG.

## Discussion

Here, we report a case of fulminant ICI myocarditis with triple M overlap syndrome refractory to multiple immunosuppressive therapies. This case highlights the life-threatening presentation of ICI myocarditis and its successful treatment with a multidisciplinary approach incorporating four administrations of abatacept. According to the recently published prognostic risk score,^5^ this patient had a score of 4 (presence of cardiomuscular symptoms, Sokolow-Lyon QRS voltage ≤0.5 mV, LVEF <50%, troponin elevation >20- to 200-fold the upper limit of normal), corresponding to an estimated 30-day risk of major cardiomyotoxic events of 81%. Although abatacept alone was insufficient to achieve complete resolution of myocardial inflammation and required additional multimodal immunosuppressive therapies, the rapid resolution of arrhythmias and conduction abnormalities, together with a reduction in TnT after the first and second doses, suggested a contribution of abatacept to clinical stabilization. Abatacept is a CTLA-4-immunoglobulin (Ig) fusion protein that inhibits T-cell co-stimulation by binding to CD80/CD86 on antigen-presenting cells, thereby blocking interaction with CD28. In a genetic mouse model with *Ctla4* haploinsufficiency and *Pdcd1* deficiency that recapitulates ICI myocarditis, CTLA-4–Ig reduced myocardial immune infiltration and improved survival,^6,7^ providing preclinical support for this approach. Although abatacept has a terminal half-life of approximately 13 days, previous studies have suggested that adequate CD86 receptor occupancy may decline within several days during the early treatment phase of severe ICI myocarditis, supporting early repeated-dosing strategies.^8,9^ In our patient, recurrent electrical abnormalities and re-elevation of TnT on Day 4 indicated persistent myocarditis activity, prompting early repeat administration. However, because serum abatacept concentrations and CD86 receptor occupancy were not measured, the pharmacokinetic basis for this deterioration remains uncertain. The optimal dosage and schedule of abatacept remain to be established, and ongoing clinical trials (NCT05335928, NCT05195645) may provide further evidence.

Another important aspect of this case was the utility of serial EMB to guide treatment decisions. Although the 2022 ESC cardio-oncology guidelines recommend EMB for haemodynamically unstable myocarditis,^4^ it carries procedural risks and may be refrained from according to the individual situation.^10^ In our patient, serial EMB provided direct histopathological information on residual myocardial inflammation that complemented biomarker monitoring. While the initial EMB supported escalation of immunosuppressive therapy, the second EMB on Day 27 demonstrated persistent CD8^+^ T-cell-predominant inflammation with CD68^+^ macrophage infiltration despite partial clinical improvement. These findings provided direct evidence of active myocarditis, supporting the continuation and further intensification of immunosuppressive therapy. Conversely, the third EMB on Day 88 demonstrated a marked reduction in myocardial inflammatory cell infiltration, supporting subsequent de-escalation of treatment. Decisions regarding continuation or intensification of immunosuppression can be challenging when based solely on biomarker trends, particularly given the potential adverse events of intensive immunosuppression and the coexistence of multi-organ irAEs. In this complex setting, direct histopathological assessment of myocardial inflammation provided additional information to the multidisciplinary team when determining treatment intensity. However, there is currently no established protocol or optimal interval for repeat EMB, and its timing must be individualized according to the clinical course, the anticipated impact of histopathological findings on management, and institutional expertise. Although no EMB-related complications occurred in this patient, this single case cannot establish the safety of repeated EMB in patients exposed to cumulative high-dose corticosteroid therapy. While this single case does not support the routine use of serial EMB, repeat EMB may complement biomarker monitoring and aid therapeutic decision-making in selected complex cases.

In conclusion, our case demonstrates the effectiveness of abatacept combined with multimodal immunosuppressive therapy in a patient with steroid-refractory fulminant ICI myocarditis and triple M overlap syndrome. Furthermore, the assessment of residual inflammation using serial EMB helps guide decisions regarding immunosuppressive therapy. Further case accumulation and prospective trials are required to establish optimal abatacept treatment strategies for this rare but life-threatening condition.

## Lead author biography


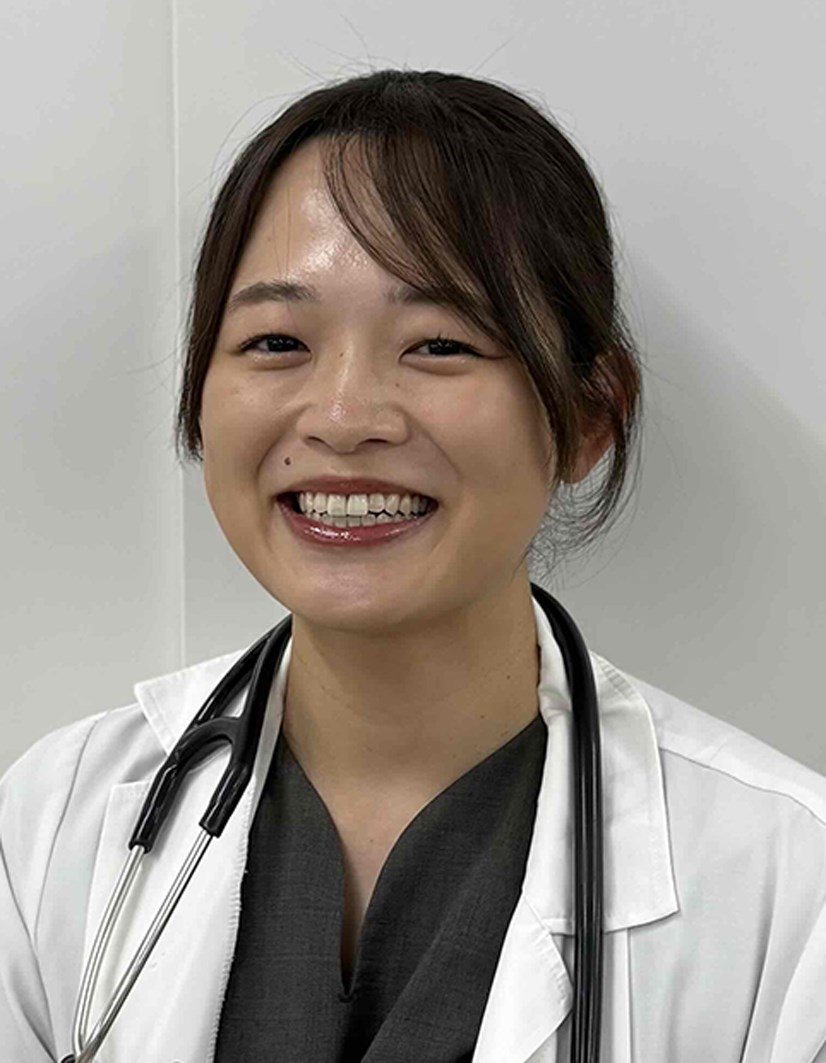
 Ruriko Matsui graduated from the University of Tsukuba, Japan, in 2022. Following her residency, she is currently a cardiology fellow at the Department of Cardiology, University of Tsukuba Hospital, and affiliated institutions.

## Supplementary Material

ytag620_Supplementary_Data

## Data Availability

Non-identifiable data underlying this article will be made available upon reasonable request to the corresponding author.
