# Supplementary material for: Abatacept for steroid-refractory fulminant immune checkpoint inhibitor myocarditis with triple M overlap syndrome: a case report with serial endomyocardial biopsy assessment
Source: Eur Heart J Case Rep. 2026 Aug 30;10(9):ytag620. doi: 10.1093/ehjcr/ytag620 (PMC13526244; doi:10.1093/ehjcr/ytag620)
Supplement: Supplementary file 1 [file ytag620_supplementary_data.zip › Supplement_fig1_ICI_EHJCR_R1_20260811_V1.1_clean.docx]

**Supplemental Figure 1. Immunohistochemical staining for CD8 and CD68 in the second and third biopsies.**

The second endomyocardial biopsy on Day 27 showed persistent CD8^+^ T-cell-predominant infiltration with CD68^+^ macrophages, whereas the third biopsy on Day 88 showed a marked reduction in inflammatory cell infiltration.

**Alt text:** The caption describes what this image shows.
